# Microbial Communities Present in Hydrothermal Sediments from Deception Island, Antarctica

**DOI:** 10.3390/microorganisms9081631

**Published:** 2021-07-30

**Authors:** Javier Vicente, Miguel de Celis, Alejandro Alonso, Domingo Marquina, Antonio Santos

**Affiliations:** Unit of Microbiology, Department of Genetics, Physiology and Microbiology, Biology Faculty, Complutense University of Madrid, 28040 Madrid, Spain; javievic@ucm.es (J.V.); migueldc@ucm.es (M.d.C.); raalonso@ucm.es (A.A.); dommarq@ucm.es (D.M.)

**Keywords:** hydrothermal sediment, metataxonomic study, microbial populations, sediment characterization

## Abstract

Deception Island is a geothermal location in Antarctica that presents active fumaroles, which confers unique characteristics to this habitat. Several studies about microbial communities in Antarctica have been carried out, nevertheless, Antarctic microbiota is still partially unknown. Here we present a multidisciplinary study about sediments obtained by deposition during 4 years in which several approaches have been considered for their characterization. First, a physicochemical characterization, using ionic chromatography and mass spectrometry for the determination of most abundant ions (chloride and sulphate) and elements (mainly silicon), was conducted. In addition, the total microbial community was studied using a metataxonomical approach, revealing a bacterial community dominated by *Proteobacteria* and *Thaumarchaeota* as the main archaeal genera and a fungal community mainly composed by *Aspergillaceae*. Culture-dependent studies showed low microbial diversity, only achieving the isolation of *Bacillus*-related species, some of them thermophilic, and the isolation of common fungi of *Aspergillus* or *Penicillium* spp. Furthermore, diatoms were detected in the sediment and characterized attending to their morphological characteristics using scanning electron microscopy. The study reveals a high influence of the physicochemical conditions in the microbial populations and their distribution, offering valuable data on the interaction between the island and water microbiota.

## 1. Introduction

Microorganisms are the most versatile and ubiquitous life form on Earth. Microbial populations can dwell and grow in extreme environments such as Antarctica. The Antarctic continent presents unique characteristics, most of its extension is ice-covered, and in some parts, different extreme conditions converge. One of these unique locations is Deception Island, which is an extremely cold location with geothermal wells.

Deception Island is a ring-shaped island of 15 km of merged diameter [1]. Mont Pond (542 m above sea level) is the highest point on the island. Deception Island is near the Antarctic peninsula, being the most active volcano in the South Shetland Islands and has been the scene of more than twenty identified eruptions over the past two centuries. The last documented eruption occurred in 1970 [2]. Bathymetric studies accomplished between 1949 and 1993 indicated a shoaling rate of 0.5 m per year, attributed to fluvial depositions and pyroclastic input [3]. It is a unique ground settled on the expansion axis on Bransfield rift [2], which divides the Archipelago from the Continent. The origin of the island is directly related to that rift when the upper part of a volcano integrated within collapsed during the Cenozoic Era.

The great majority of Antarctic volcanoes do not present any evidence of geological activity, and only four stand out as hydrothermal habitats in Antarctica. Three of them are in the continent, in Victoria Land, and the fourth is Deception Island [4]. Those Antarctic geothermal locations are small areas of relatively hot liquid water in a vast, dry, and cold territory, composed of mineral soils like lapilli or pyroclastic ash [5]. Those locations are upon regions that present raised temperatures near hydrothermal vents or cracks related to geological active volcanoes. In Deception Island, numerous hot soils, hot springs, and fumaroles evidence volcanic activity. The abundant geothermal activity provides the appropriate characteristics to the development of a singular environment [6].

Deception Island is one of the locations that has contributed to a greater extent of the knowledge about hydrothermal ecosystems in Antarctica because, since 1930, it has accommodated numerous scientific stations due to its accessibility [5]. 

The whole experimental terms offered by this habitat allow the presence of microbial communities that are extremely adapted to the ground [7]. The communities are locally adapted, responding to physiochemical gradients and biotic characteristics of soils [8]. The first study of the Antarctic soil microbiology was carried out by Darling and Siple [9], that, together with other studies [10], described different microbial species. *Bacillus subtilis* and *B. megaterium* were the first species described in the Antarctic continent. Subsequent researchers indicated an apparent microbial poverty of Antarctic soils, some of them being described as sterile [11]. Probably, in those cases in which any viabilization was achieved, the samples were composed only of viable but non-culturable microorganisms [12].

The 21st century, by applying different molecular approaches, was when the real microbial abundance and diversity present in Antarctic soils was made known [13]. The most common bacterial phyla in island-soils are *Proteobacteria, Bacteroidetes,* and *Actinobacteria* [14,15], which are common to those observed in other regions of the continent, which communities are mainly composed of *Actinobacteria, Proteobacteria, Bacteroidetes, Acidobacteria,* or *Deinococcus-Thermus* [8]. As far as fungi are concerned, few studies have been carried out; some of them [16,17] demonstrated that the community is mainly composed of *Ascomycetes*. Nevertheless, despite these studies, the microbial community is still poorly characterized, representing a huge unknown potential of biotechnological applications.

The uniqueness of this study lies in the characteristics of the samples; the sediment has not been taken in a specific moment, but by the accumulation over four years. During this time, the material that seawater currents carry in suspension has been accumulated in the precipitation column used for sampling. This study aims to gain a deeper insight into the Polar microbiology, by describing the microbiome of the sediments generated in a unique location on Deception Island. We analyze the nature of the sediments, the microbial input on the Antarctic continent, and the settlement of new microbial populations.

## 2. Materials and Methods

### 2.1. Sampling

The sample object of study was taken from the hydrothermal precipitation column number 2 from the HYDRODEC-2000 campaign (Special Action Project ANT1998-1557-E/HESP from the Spanish Antarctic Program). The column was placed in Deception Island, South Shetland Islands, Antarctica (60°34′9.300″ W–62°58′48.30″ S), Antarctic Specially Protected Area 140 (ASPA 140), in an intermediate point between Kroner Lake and Whalers Bay coast, and in a shallow coastal zone in which the temperature is between 40 and 60 °C according to previous works [1,4]. The sediments in the column were obtained by deposition from the upper zone of material dragged by sea currents and tides for four years. After column removal, sediments were stored at −20 °C until laboratory processing.

### 2.2. Geological Study and Determination of Physicochemical Parameters

#### 2.2.1. Ionic and Atomic Compositional Analysis: Inductively Coupled Plasma Atomic Emission Spectroscopy (ICP-OES) and Ionic Chromatography (IC)

For the aqueous extract from the sample, five grams of sediment were resuspended in 50.0 milliliters of MilliQ water and kept at room temperature and constant shaking for 24 h. The mixture was centrifuged, and the supernatant was recovered and filtered by a 0.45 microns PES filter (Whatman, Maidstone, Kent, UK). MilliQ water was added up to 100 milliliters. An ionic chromatograph with a conductivity detector, 940 Professional Ionic Chromatograph Vario One (Metrohm, Herisau, Switzerland), and a MetroSep A Supp 7 (250.0 × 4.0 mm) column were used. Fifty microliters of the sample were used in a mobile phase of Na_2_CO_3_ and a constant flux of 0.70 mL·min^−1^. The sample was analyzed in triplicate.

For the ICP-OES study, 0.25 g of sediment were resuspended in 5.0 of nitric acid. The mixture was kept at 100 °C up to 24 h. Therefore, 5.0 milliliters of fluorhydric acid was added for silicate digestion, and the mixture was kept at 100 °C for 24 h. Finally, perchloric acid was added and the mixture was maintained on a heating plate until it dried. The product was carried up to 50 milliliters with MilliQ water, and the final concentration of nitric acid was 8%. A SPECTRO Arcos spectrometer was used and analyzed in triplicate. International Reference Material CRM-277 Estuarine Sediment was used as standard for comparison. 

Both analyses were carried out at the Geologic Techniques and Archaeometry Research Assistance Centre of the Geological Sciences Faculty at the Complutense University of Madrid.

#### 2.2.2. Structural Analysis by X-ray Powder Diffraction (XRD)

The sample was ground on an agate mortar and passed through a 52-micron sieve. A powder coat was spread on an aluminum slide and placed on a D500 Siemens diffractometer with a graphite monochromator. The analysis was carried out using a cupper Kα anode (λ = 1.54 Å). An X-ray generator was set to an acceleration voltage of 40 kV and a filament emission of 30 mA. The exploration angle was set between 2 and 70° of 2-Theta, and the speed was set at 2° per minute. The diffract Plus diffraction program and EVA 9 (Siemens, Munich, Germany) were used as analysis software. Minerals were identified using the standard reference diffractograms acquired from the American Society for Testing and Materials (ASTM) database.

The analysis was carried out at the Mineralogy and Crystallography Department of the Geological Sciences Faculty at the Complutense University of Madrid.

### 2.3. Microbial Diversity Analysis

#### 2.3.1. Metataxonomic Study

The sediment sample was analyzed following a 16S and ITS metabarcoding strategy for determining prokaryotic and fungal populations. The sample was stored at −20 °C until DNA extraction was performed. DNA extraction was carried out using 0.25 g of the sample and using a commercial kit (DNeasy Powerlyzer Powersoil Kit, Qiagen, Hilden, Germany). The V4 region of the 16S rRNA gene was amplified by PCR, using primers 515F (GTGYCAGCMGCCGCGGTAA) and 806R (GGACTACNVGGGTWTCTAAT) and the ITS1 region using WineSeq custom primers (patent number: Patent WO2017096385). Libraries were prepared following the two-step PCR Illumina^®^ protocol, applying the Nextera XT index kit (Illumina Inc. San Diego, CA, USA), as described. Then, these were subsequently sequenced on Illumina^®^ MiSeq instrument (Illumina^®^, San Diego, CA, USA) using 2 × 300 paired-end reads. PCR conditions such as the number of cycles, annealing temperature, thermocycler, and Master-mix composition were carried out according to the WineSeq^®^ technology procedures [18]. The resulting fastQ sequences were analyzed following the DADA2 pipeline implemented in R using default parameters [19]. This pipeline implements an error correction model, allowing the differentiation of a single nucleotide [20], giving as a final output an Amplicon Sequence Variants (ASV) table. The taxonomic assignment was performed using the naïve Bayesian classifier implemented in DADA2 using Silva (release 132) as a reference database [21] for prokaryotes and the UNITE reference database for fungi [22] with a bootstrap cut-off of 80%.

#### 2.3.2. Isolation and Identification of Viable Microorganisms

Different culture broths were used to isolate different taxa of bacteria and fungus: Tryptone Soy Broth (TSB, Conda–Pronadisa Laboratories); Potato Dextrose Broth, (PDB, Conda–Pronadisa Laboratories, Madrid, Spain); Yeast Malt Broth, (YMB, Conda–Pronadisa Laboratories, Madrid, Spain); and Sea Water Yeast Extract (SW); 9K and R2A [23]. Sediment was resuspended in the culture media in a 1:10 ratio on sterile 100 mL Erlenmeyer flasks and incubated at 15, 32, and 60 °C with constant shaking (120 rpm). 

Every 24 h, up to 96 h, 100 µL aliquots were sampled and spread on the correspondent solid medium and incubated under the same temperature conditions. Attending to their morphological characteristics, different colonies were picked and streaked on agar plates. Axenic cultures were maintained in glycerol (20%) at −80 °C.

DNA extraction was carried out according to the Cenis modified method, using silica spheres (0.2–0.5 mm diameter) for cellular breaking [24]. Identification was conducted according to the 16S ribosomal (for bacteria) region and ITS region (for fungi) using the extracted DNA. Y1 (5′TGGCTCAGGACGAAGCTGGCGGC3′) and Y2 (5′CCTACTGCTGCCTCCCGTAGGAGT3′) were the primers used for amplification of the 16S region. PCR conditions, up to 30 cycles, were: denaturing, 95 °C, 45 s; annealing, 58 °C, 1 min; extension, 72 °C, 45 s. NL1 (5′GCATATCAATAAGCGGAGGAAAAG3′) and NL4 (5′GGTCCGTGTTTCAAGACGG3′) were the primers used for the amplification of the ITS region. PCR conditions, up to 30 cycles, were: denaturing, 95 °C, 1 min; annealing, 56 °C, 90 s; extension, 72 °C, 2 min. The PCR reaction mixture was: 25 µL DreamTaq Green DNA polymerase 2x (Fisher Scientific, Waltham, MA, USA), 2 µL of each primer (50 µM), 2 µL of template DNA, and 19 µL of molecular grade water.

PCR amplicons were visualized on a TAE-agarose 1% gel with GelRed^®^ (2 µL/30 mL) and then purified using the mi-PCR Purification Kit (Metabion, Planneg, Germany). Fragments were Sanger-sequenced (Macrogen Europe, Amsterdam, Netherlands) using an ABI3730 XL Sanger technology sequencer. Sequences were compared to GenBank sequences using the Basic Local Alignment Search Tool (BLAST) from the National Center for Biotechnology Information (NCBI).

#### 2.3.3. Phenotypical Characterization of the Bacterial Isolates

Growth of the thermophilic strains was verified at 45 and 75 °C using SW as the culture media and measured turbidically at 600 nm. Salinity resistance characterization of the bacterial isolates was determined using Sea Water Yeast Extract agar plates with different marine salt concentrations (0 to 16% through 2% growths). The extracellular enzymatic characterization was performed using the Ashby for nitrogen-fixation activity, Pikovskaya for phosphate-solubilizing activity [25], Aleksandrow for potassium-solubilizing activity [26], and TSA supplemented with 10 g/L of starch (Sigma-Aldrich, Burlington, MA, USA) for amylolytic activity or 10 g/L gelatin (Sigma-Aldrich, Burlington, MA, USA) for protease activity determination. Fifty microliters of a bacterial suspension (0.5 McFarland scale) was spotted onto the plates. Incubation was carried out at 32 and 60 °C, depending on the isolation temperature, up to 48 h. Starch-TSA and gelatin-TSA were revealed with Lugol and Frazier reagents, respectively.

#### 2.3.4. Study of Scanning Electron Microscopy (SEM)

The analysis was carried out at the Geologic Techniques and Archaeometry Research Assistance Centre of the Geological Sciences Faculty from the Complutense University of Madrid. The sample was placed on a carbon tape, gold-coated, and visualized using a JEOL JSM-820 scanning electron microscope (SEM). For morphological identification of diatoms, SEM digital images were compared with online databases [27,28,29]. We discriminated between central and pennal divisions; and, as morphologic differential characteristics, we used the apical, transversal, and pervalvar ratios, as well as ornamentation characteristics. 

## 3. Results

### 3.1. Geological Study and Determination of Physicochemical Parameters

Ionic chromatography (IC) analysis showed that chloride and sulphur are the main ions in the sample (Table 1), whereas the atomic composition determinations (ICP-OES) indicated that silicon, aluminum, and iron are the main macroelements, and, regarding the microelements, strontium, sulphur, and nickel are the commonest (Table 2).

**Figure A1:**
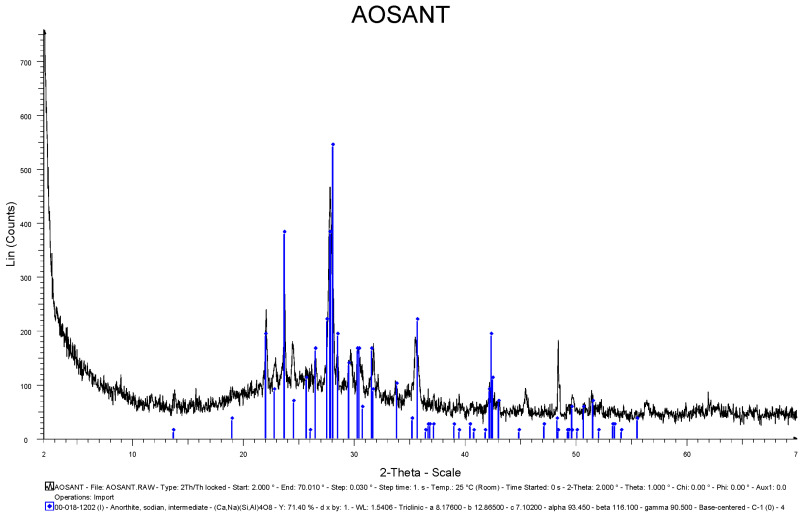
X-Ray Diffractogram showing the anorthite profile according to ASTM file number 00-018-1202.

Using XRD, we could identify two principal minerals in the sediment sample, anorthite and halite, identified using the American Society for Testing and Materials (ASTM) file number 00-018-1202 and 01-075-0306, respectively. The sample presented a very low crystalline structure, conformed by amorph glasses due to the shape; it showed numerous not well-defined peaks and a central bulge standing out (Appendix A, Figure A1).

### 3.2. Microbial Diversity Analysis

#### 3.2.1. Metataxonomic Study

**Figure 1:**
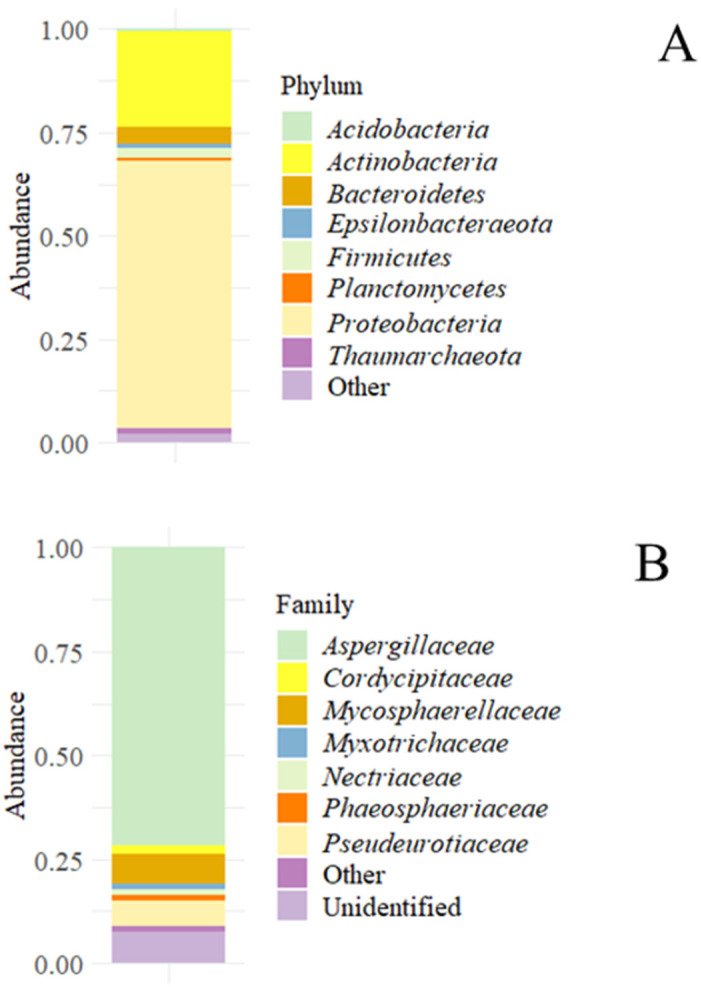
Accumulated microbial abundances in the sediment sample. (**A**) Accumulated bacterial and archaeal abundances at the phylum level. (**B**) Accumulated fungal abundance at the family level.

The fastQ sequences analyzed in R provided a total of 70.287 good quality reads for prokaryotes and 86.890 for fungi (NCBI Accession Number PRJNA702109). Considering prokaryotes, at a phylum level, the most abundant populations detected were *Proteobacteria* (more than 50% of the total reads) and *Actinobacteria* (around 25%); furthermore, some Archaea, as *Thaumarchaeota*, were also found (Figure 1A). Regarding the fungal populations found in the sample, they were dominated by almost 75% at the family level by *Aspergillaceae*; the remaining the population was formed by *Phaeosphaeriaceae*, *Pseudeurotiacee,* and other unidentified families (Figure 1B).

#### 3.2.2. Isolation and Identification of Viable Microorganisms 

To determine the viability of the meta-taxonomically detected populations, an isolation study conducted after an enrichment step in different broths was conducted for fungi and bacteria. No bacteria were isolated when the incubation was conducted at 15 °C; however, most isolates were isolated at 32 °C, and a few at 60 °C (Table 3). Concerning the bacterial isolates, a total of 30 strains were isolated, all of them identified as Bacillus-related species.

Only 12 fungal isolates (Table 4) were obtained after enrichment, most of them belonging to the genus *Penicillium*, as indicated by molecular identification.

#### 3.2.3. Phenotypical Characterization of the Bacterial Isolates

The bacterial strains of the present study were diverse according to their phenotypical characteristics (extracellular enzyme profiles and salinity resistance). All isolates were able to grow in saline concentrations up to 8% *w*/*v*. From the 30 bacterial isolates tested, 25 were able to grow at 10% NaCl, 15 at 12%, 11 at 14%, and 9 at 16%.

The study of a basic panel of metabolic traits of the isolates indicated that only one bacterial isolate (DIP-7) was able to grow in a nitrogen-free medium, indicating that it was a free-living nitrogen-fixing strain. Furthermore, only one strain was found to be able to solubilize potassium (isolate DIP-18). More common among the isolates were the phosphate solubilizing bacteria (near 73%) which are very important for biogeochemical cycles, increasing the P availability in soils. Finally, proteolytic (near 67%) and amylolytic activities (60%) were normal among the isolates.

#### 3.2.4. Study of Scanning Electron Microscopy (SEM)

**Figure 2:**
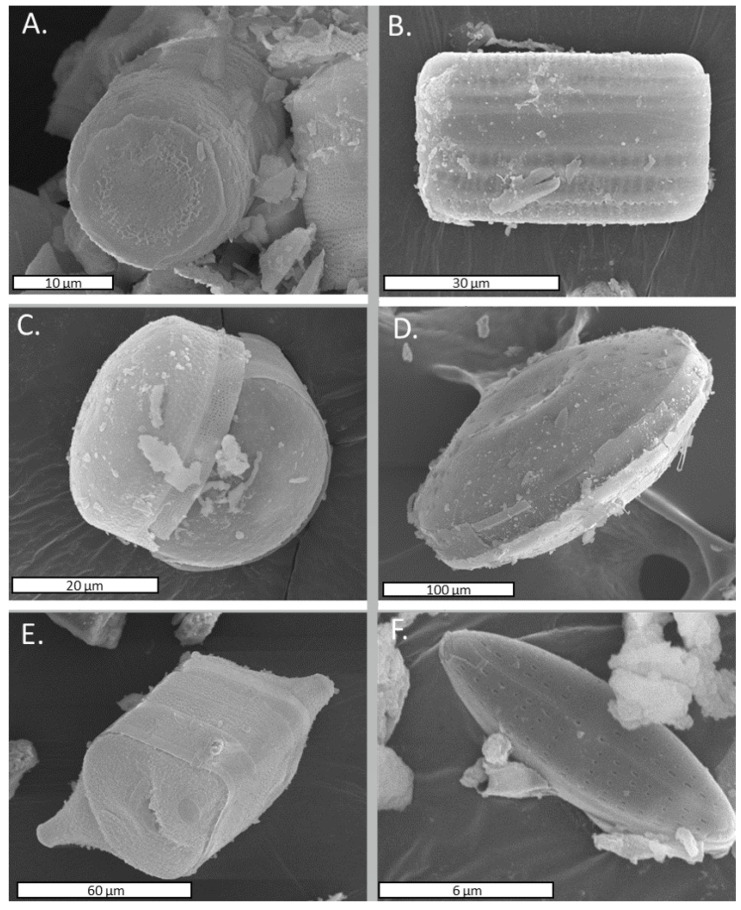
Identified diatoms present in the sediment according to the morphological characteristics observed by SEM. (**A**) *Dactyliosolen* spp., (**B**) *Thalassiosira* spp., (**C**) *Coscinodiscus* spp., (**D**) *Actynocyclus* spp., (**E**) *Odontella* spp., (**F**) *Psammothidium* spp.

The microscopic analysis of the sediments revealed that diatoms were present in the sediment at a high frequency. Up to six different species of diatoms were found according to the study of their morphological characteristics observed by SEM: *Dactyliosolen* spp., *Thalassiosira* spp., *Coscinodiscus* spp., *Actynocyclus* spp., *Odontella* spp., and *Psammothidium* spp. (Figure 2).

## 4. Discussion

Deception Island is covered by lapilli and pyroclastic ash on most of its surface [30]. The DRX study of the sample revealed two main minerals, anorthite and halite. Anorthite could be explained by the presence of lapilli since it is formed by basaltic-andesitic and volcanic glasses. The andesite that conforms lapilli is fundamentally composed by plagioclases, belonging to the albite-anortite series, from the tectosilicates group which their general formula is (Na, Ca) (Si, Al)_3_O_8_. Halite, another main component of the sample, comes from the deposition of marine water.

Recently, some DRX studies of samples coming from Deception Island have been carried out. Lezcano and colleagues analyzed a soil sample from Cerro Caliente Hill and determined that the phyllosilicates (montmorillonite, nontronite, and saponite) group was the most prevalent group of minerals [31]. Nevertheless, regarding the chemical composition of the sediments, some ions differed in that study compared to our findings: in the case of soluble SO_4_^2−^, the observed concentration was one thousand times lower (1.39 ± 1.17 μg/g), and NO^3−^ was ten times less concentrated (0.22 ± 0.15 μg/g). The chemical analysis of several sediment samples revealed that in Whaler’s Bay some metallic elements were more concentrated than in other island locations [32]. Among all, Fe was observed as the most common element (15.814 ± 581 μg/g).

Regarding other kinds of substrates, the chemical composition is more different. The ionic analysis of melted water [33] revealed quite a different composition if compared to those obtained in geothermal sediments: the concentration of NO^3−^ rise up to 3 mg/mL, and other elements, like S (6.76 ppm compared to 329 ppm), on the contrary, were less concentrated. Some of them, like Mg (0.1 ppm), were in similar concentrations.

Our samples were formed by sediments that air and water transported and then, were deposited. The chemical analysis carried out in this study showed that the sediment is mainly composed of materials that conform to the island, probably originating there. Nevertheless, the ionic composition is slightly different from the sediments obtained in the island and glacial water. The main difference is in regard to ions such as SO_4_^2−^ and NO^3−^. In the case of nitrates, our sediments could have been enriched by materials from glaciers. Due to the presence of the fumaroles, a soil–temperature gradient was established as the temperature increases, and glaciers melt enriching the seawater. On the contrary, we could hypothesize that sulfur does not come neither from the island nor from glaciers, in both cases, it is present in much less concentration. High concentrations of sulphur could be found due to the presence of fumaroles and volcanic activity [34]. An indicator of volcanic enrichment is the presence of high concentrations of iron, manganese, and silicon at high concentrations [35]. In this sample, we cannot affirm that a volcanic enrichment could have occurred because sulphur and iron concentrations are not in the proper ratio. Silicon cannot be considered an indicator of volcanic activity in this case either, this element may come from materials that conform to the island. Either way, geothermal phenomena are not only the emission of volcanic materials through steam vents or fissures.

We can highlight the presence of amorphous minerals detected by SEM-EDX. These minerals, composed of sulphur trioxide and aluminum oxide, could be interpreted as particles of the sediment coming from the volcanic activity that occurred in the last century [2].

Other interesting and abundant elements detected in the optical emission spectroscopy studies were silicon, aluminum, nickel, sodium, calcium, and strontium, which are the main elements of basalt and andesite [36].

In conclusion, we can assume that the origin of the materials present in the sample are sediments that come from the island dragged by water.

Our bacterial meta-taxonomic results are comparable to previously reported results [36], showing a stable bacterial community in different sediment samples at Whaler’s Bay over several years. In that study, *Proteobacteria*, *Bacteroidetes,* and *Actinobacteria* were the main groups. In other analyzed substrates, the results are similar. The microbial composition of different microbial structures such as biofilms and microbial mats have been studied. *Proteobacteria* is the most common phylum (in around 75%) in different bacterial biofilms isolated at Whaler’s Bay [37], as well as in microbial mats taken from the island (in around 30%). *Bacteroidetes* and *Acidobacteria* (in different ranges depending on the microbial mat) are also usually present [31]. Martínez-Alonso and colleagues studied the volcanic endoglacial sediments and described *Actinobacteria* as the most common phylum in this ecosystem (30%), followed by *Bacteroidetes* (27%) and *Proteobacteria* (15%) among others [33].

The archaeal phylum *Thaumarcheota* detected in the meta-taxonomic analysis has been yet to be described on the island. Lezcano and colleagues described this as the most common phylum in different microbial mats, representing near 35% of the archaeal community [30].

To study the viable microbial population present in the sediment sample and their adaptations to such an environment, an isolation protocol was conducted. It should be highlighted that the great difference observed between the present microbiota, analyzed using molecular approaches (meta-taxonomic study), and the viable microbiota, by cultivation on agar plates, is known as the “the great plate count anomaly”. Nevertheless, this low percentage of viabilization has been previously reported in similar Antarctic samples from other studies [6]. Using culture media similar to those previously used in other studies, both for chemoheterotrophs and chemolithotrophs, the majority of isolates presented a chemoheterotrophic metabolism [6,14]. All the isolated strains belonged to the *Bacillus*-related genera, which are spore-forming species, allowing survival under adverse conditions. Furthermore, only 7.69% of the species detected by the meta-taxonomic analysis have been described as spore-forming microorganisms. The absence of lithotrophic isolates could be explained by these two facts: the lack of endospore-forming species (which affects the long-term survival of the community), and the nutritional requirements.

Studies carried out during the 20th century in soils with different characteristics, discovered bacteria that could only be identified up to the genus level and, interestingly, all of them belonged to the *Bacillus* genus [11,38]. Later, advances in molecular techniques for microbial identification and taxonomy made possible the new reidentification of these isolates, describing new genera such as *Alicyclobacillus* [39] and *Geobacillus* [40].

Thermophilic strains of *Geobacillus* spp. and *Brevibacillus thermoruber* where previously described at Deception Island by Muñoz and colleagues in a sample from Fumaroles Bay, located northwest from our sampling point [41]. Other species isolated in this research, such as *Bacillus cereus* and *B. megaterium*, have been described previously on Deception Island [42]. More recently, some thermophilic strains have been isolated from the island, despite sediment temperature from Whaler’s and Fumarole Bays, either from glacier or fumarole zones [14]. The isolates belonged to *Bacillus*-related genera such as *Geobacillus*, *Brevibacillus* and *Anoxybacillus,* and were recovered from samples with environmental temperatures ranging from 0 to 80 °C and using general culture media, such as TSA or Marine Agar, such as in our study.

High saline resistance of the isolated strains could be an adaptation to the dehydration caused under freeze and high salinity conditions [43]. This could be related to wider resistance to several stresses [44]. Our strains were halotolerant, all of them were able to grow in up to 8% NaCl (*w*/*v*) and some of them up to 16%. Some authors have reported osmophilic bacterial strains, isolated from the rhizosphere of Antarctic plants, resistant to NaCl concentrations up to 16% [45]. This generalized osmotolerance is linked with molecular and physiological adaptations to these environments as indicated by [36] that analyzed the stress response genes in Whaler’s Bay, describing the osmotic stress genes as the most common stress genes in a metagenomic analysis.

The production, transformation, decomposition, and/or transport of organic matter is a central part in the geochemical cycle of bioelements. In that way, microorganisms play a fundamental role, contributing an efficient set of extracellular enzymes to an optimum nutrient uptake [45]. In our study, the strains presented different enzymatic profiles, with few strain-specific activities. Phosphatase, protease, and amylase were the commonest in our study as was also reported in other studies [46].

The studies regarding the fungal diversity on the island reported the same overall pattern of dominant fungal populations by isolation and meta-taxonomic approaches. Concerning the cultivable fungal community, we have detected a low diversity level as other studies have shown [16,47], with most of the isolates identified as members of the *Aspergillus* and *Penicillium* genera. These genera have been isolated regardless of the soil temperature, both in hot and cold volcanic soils of the island [16]. Only one isolate belonged to a different genus, *Exophiala*, nevertheless, this was usually found in the meta-taxonomic studies carried out in the same island [17]. *Exophiala* spp., as well as some *Penicillium* spp., have been described to be extremely thermotolerant [48]. In other locations, such as King George Island (South Shetland Islands), *Penicillium* spp. has also been frequently isolated [49].

According to the reported results, more than 1000 fungal species have been described in morphological or cultivation studies of Antarctic fungi, nevertheless, the use of ‘omic’ approaches is essential in describing the microbial life in each habitat [17]. Several ‘omics’ (metabarcoding or metataxonomic) studies have been realized. In all of them [17,31,50], *Ascomycetes* was the dominant phylum. Our metataxonomic data suggests that the fungal community is dominated by the *Aspergillaceae* family (*Ascomycetes*). The meta-barcoding analysis of fungal diversity in different areas (impacted and non-impacted locations) of the island revealed that the main phylum is *Ascomycota*, up to 44.3% of the ASVs, not being influenced by the consideration or protection status of the area [17]. In other types of samples, as well as in soils, the population was dominated by *Ascomycota*.

In microbial mats, sampled at different temperatures, *Ascomycota* was the predominant fungal phyla among all the eukaryotes. At 88 °C, this phylum was the most abundant (9% of the phyla) as well as at 2 °C (2%); interestingly, at 8 °C it practically disappeared (abundance less than 0.55%) due to an increase of the eukaryotic-autotroph population [30].

As indicated before, a diatom population was also present in the sediment, and it was described using the morphological data obtained with SEM. Only the *Psammothidium* and *Thalassiosira* genera, two out of the six diatom genera, have been previously described on the island, near Kroner Lake [51]. The remaining genera (*Dactyliosolen, Coscinodiscus, Actynnocyclus,* and *Odontella*) have not been previously described on the island, but their presence on the continent has already been documented. Generally, benthonic diatoms belong to the pennate group, whereas those in the pelagic habitat belong to central ones [52]. In this study, most of the identified genera were identified as central ones and were probably diatoms (or their cellular rests) with a pelagic habitat that were dragged to the sampling point by sea-water streams.

In this study, the different analyses carried out in such a unique sediment sample provided interesting information about the microbiological and physicochemical influences between the island and the surrounding sea. The ionic and mineral composition revealed the confluence between the sea and glacier water, creating a transition zone between ice, land, and sea. The microbiological determinations provided interesting information about the community present in that confluence zone. The metataxonomic analysis itself could only show the quantitative/qualitative structure of the community, but its comparison to other types of samples could give valuable information about the ecological micro-structure and interactions between two different environments, defining different roles and influencing mechanisms. In addition, the viable community inhabiting such an extreme environment showed different metabolic potentials, not only in their enzymatic activities but also in their resistance profiles to environmental stresses, promising a high biotechnological potential capable of being screened for useful industrial enzymes or bioactive compounds.

## Figures and Tables

**Table 1:** Main ions present in the sediment sample determined by ionic chromatography.

Ion	Concentration (μg/g)
Cl^−^	4075 ± 408
SO_4_^2−^	1009 ± 202
Br^−^	14 ± 1
NO_3_^−^	6 ± 1
F^−^	0.42 ± 0.04

**Table 2:** Main elements present in the sample determined by ICP-OES.

Elements	Percentage (%)	Trace Elements	Amount (μg/g)
Si	17.49 ± 0.71	Sr	351.64 ± 43.84
Al	8.14 ± 0.35	S	329.27 ± 40.31
Fe	6.34 ± 0.07	Ni	172.00 ± 0.00
Na	3.4 ± 0.00	Zn	87.98 ± 2.83
Ca	2.29 ± 0.17	Cu	55.32 ± 6.36
K	0.77 ± 0.01	Ba	52.21 ± 7.18
P	0.17 ± 0.01	Co	45.99 ± 1.41
Mn	0.11 ± 0.00	Li	18.89 ± 2.82
Mg	0.11 ± 0.03	Mo	17.66 ± 7.76
Ti	0.07 ± 0.00		

**Table 3:** Isolated bacterial strains in the sediment. Culture media and temperature of isolation.

Isolates	Identification	Enrichment Media	Temperature (°C)	Enzymatic Activity	Salinity Resistance	NCBI Accesion Number
DIP-1	*Bacillus* sp.	SW	32	-	8	MZ600240
DIP-2	*Bacillus* sp.	SW	32	-	8	MZ600241
DIP-3	*Bacillus* sp.	SW	32	AMIL, PROT	10	MZ600242
DIP-4	*Bacillus* sp.	SW	32	PHOS	8	MZ600243
DIP-5	*Bacillus* sp.	SW	32	-	8	MZ600244
DIP-6	*Bacillus* sp.	SW	32	PROT	16	MZ600245
DIP-7	*Bacillus* sp.	SW	32	NIT, PHOS, AMIL, PROT	10	MZ600246
DIP-8	*Bacillus* sp.	SW	32	-	16	MZ600247
DIP-9	*Bacillus* sp.	SW	32	PHOS	10	MZ600248
DIP-10	*Bacillus cereus*	SW	32	PHOS, AMIL, PROT	12	MZ600249
DIP-11	*Bacillus* sp.	SW	32	PHOS, AMIL, PROT	10	MZ600250
DIP-12	*Bacillus* sp.	SW	32	PHOS, AMIL, PROT	16	MZ600251
DIP-13	*Bacillus megaterium*	SW	32	PHOS, AMIL, PROT	10	MZ600252
DIP-14	*Bacillus* sp.	SW	32	PHOS, AMIL, PROT	16	MZ600253
DIP-15	*Bacillus* sp.	SW	32	PHOS, AMIL, PROT	12	MZ600254
DIP-16	*Bacillus simplex*	SW	32	PHOS, AMIL, PROT	10	MZ600255
DIP-17	*Bacillus* sp.	SW	32	PHOS, PROT	10	MZ600256
DIP-18	*Bacillus* sp.	SW	32	PHOS, POT, AMIL, PROT	16	MZ600257
DIP-19	*Bacillus megaterium*	SW	32	PHOS, AMIL, PROT	16	MZ600258
DIP-20	*Bacillus* sp.	SW	32	PHOS, AMIL, PROT	16	MZ600259
DIP-21	*Bacillus mycoides*	SW	32	PHOS, AMIL, PROT	12	MZ600260
DIP-22	*Bacillus simplex*	SW	32	AMIL	16	MZ600261
DIP-23	*Bacillus circulans*	SW	32	PHOS, AMIL, PROT	14	MZ600262
DIP-24	*Bacillus aryabhattai*	SW	32	PHOS, PROT	16	MZ600263
DIP-25	*Bacillus* sp.	SW	32	PHOS, AMIL, PROT	14	MZ600264
DIP-26	*Bacillus* sp.	SW	32	PHOS, AMIL, PROT	10	MZ600265
DIP-27	*Bacillus* sp.	SW	32	PHOS, AMIL, PROT	12	MZ600266
DIP-28	*Brevibacillus thermoruber*	SW	60	PHOS	12	MZ600267
DIP-29	*Geobacillus* sp.	SW	60	-	10	MZ600268
DIP-30	*Bacillus* sp.	SW	60	PHOS	10	MZ600269

AMIL: amilase; PROT: protease; PHOS: phosphatase; NIT: nitrogen fixation.

**Table 4:** Fungal strains isolated in the sediment. Culture media and temperature of isolation.

Isolates	Identification	Enrichment Media	Temperature (°C)	NCBI Accesion Number
DIF-1	*Aspergillus* sp.	PDB	15	MZ602115
DIF-2	*Penicillium chrysogenum*	YMB	15	MZ602116
DIF-3	*Aspergillus* sp.	PDB	15	MZ602117
DIF-4	*Aspergillus sydowii*	PDB	15	MZ602118
DIF-5	*Penicillium* sp.	PDB	15	MZ602119
DIF-6	*Penicillium* sp.	YMB	32	MZ602120
DIF-7	*Penicillium* sp.	PDB	32	MZ602121
DIF-8	*Penicillium crustosum*	PDB	32	MZ602122
DIF-9	*Exophiala* sp.	PDB	32	MZ602123
DIF-10	*Penicillium chrysogenum*	PDB	32	MZ602124
DIF-11	*Penicillium* sp.	PDB	32	MZ602125
DIF-12	*Penicillium crustosum*	YMB	32	MZ602126
